# Interaction Proteomics of Polycystins 1 and 2 Reveal a Novel Role for the BLOC-1/BORC Lysosomal Positioning Complex

**DOI:** 10.1016/j.mcpro.2025.101091

**Published:** 2025-10-12

**Authors:** Fatima Lukmani, Jonathan M. Shillingford, Mackenzie Brauer, Dhairya Pancholi, Jonathan St- Germain, James A. Shayman, Brian Raught, Gagan D. Gupta

**Affiliations:** 1 Department of Chemistry and Biology, Toronto Metropolitan University, Toronto, Ontario, Canada; 2 Department of Internal Medicine, University of Michigan Medical School, University of Michigan, Ann Arbor, Michigan, USA; 3 Department of Medical Biophysics, University of Toronto, Toronto, Ontario, Canada

**Keywords:** polycystic kidney disease, polycystins, proximity proteomics, lysosome signaling, interaction network

## Abstract

*PKD1* and *PKD2* are the most commonly mutated genes in autosomal dominant polycystic kidney disease (ADPKD). However, the precise roles of the encoded polycystin 1/2 (PC1 and PC2) proteins, and how their functions are disrupted in ADPKD, remain unclear. Here, we characterize the protein interaction networks of PC1 and PC2 in cycling and ciliated cells using proximity-dependent biotinylation (BioID), identifying a common set of 172 proteins that interact with the C terminus of PC1 and the full-length PC2 protein, enriched in autophagy regulators, endoplasmic reticulum tethers, endoplasmic reticulum stress proteins, and other proteins previously linked to ADPKD. Notably, we also find that PC1 specifically interacts with ciliary and lysosomal proteins, including components of the biogenesis of lysosome-related organelles complex (BLOC-1) and BLOC-one-related-complex (BORC). BLOC-1/BORC colocalizes with PC1 at lysosomes and cilia and is required for proper ciliary PC1 localization. In addition, PC1 mutant kidney cells derived from an ADPKD patient display defects in BLOC-1/BORC distribution. Renal cells depleted of PC1 exhibit abnormal lysosomal distribution, similar to those depleted of BLOC-1/BORC components. Finally, shRNA knockdown of BLOC-1/BORC components promoted cystogenesis in a 3D *in vitro* cyst model, and this could be attenuated by heterologous expression of the C terminus of PC1. This rich dataset thus links the BLOC-1/BORC complex to PC1 function and can be further mined for additional mechanistic insights into the PC1/2 ADPKD proteins.

## Highlights

• Proximity-dependent biotinylation mapping of the C terminal tail of polycystin 1, and full-length polycystin 2, identifies a rich dataset enriched in lysosomal and autophagy regulators, ER tethers, and ER stress proteins.
• Polycystin 1 interacts with members of the BLOC-1/BORC complexes, and these colocalize with PC1 at lysosomes and primary cilia.
• BLOC-1/BORC components are required for proper ciliary localization of PC1, and their lysosomal distribution is altered in an ADPKD patient cell line.
• Depletion of BLOC-1/BORC components promotes cystogenesis in 3D culture models, which can be attenuated by expression of the PC1 C terminus.

## Graphical Abstract

**Figure:**
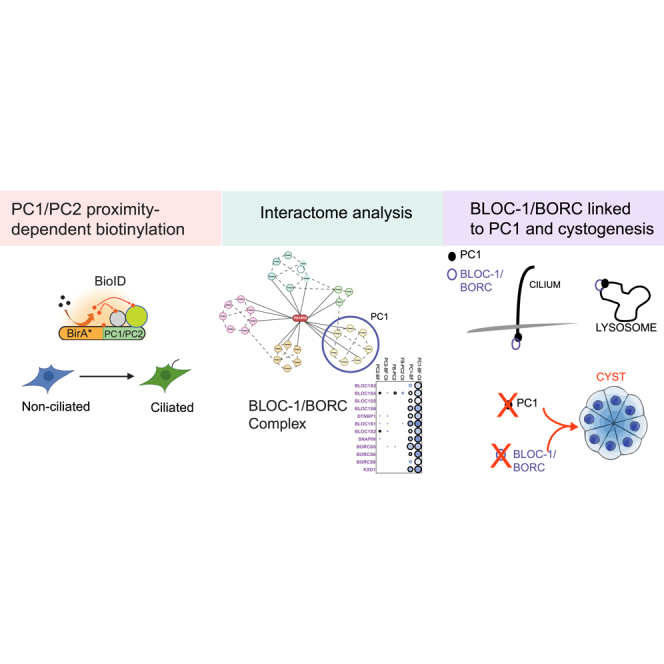

## In Brief

Lukmani *et al*. employed proximity-dependent biotinylation to define the interaction networks of polycystin 1 and 2, the proteins most frequently mutated in autosomal dominant polycystic kidney disease. They identified 172 common interactors enriched in autophagy and ER stress pathways, and discovered that PC1 specifically associates with BLOC-1 and BORC complexes at lysosomes and cilia. Depletion or deletion of BLOC-1/BORC components disrupts PC1 ciliary localization. Furthermore, an ADPKD patient cell line exhibits altered lysosomal distribution of BLOC-1/BORC components. Depletion of BLOC-1/BORC components in a renal cyst model results in enhanced cystogenesis, revealing a previously unrecognized role for these lysosomal trafficking complexes in polycystic kidney disease pathogenesis.

Autosomal dominant polycystic kidney disease (ADPKD), a prevalent and potentially lethal monogenic disorder, is characterized by progressive renal cyst formation driven by mutations in *PKD1* or *PKD2*, encoding polycystin-1 (PC1) and polycystin-2 (PC2), respectively (1). PC1 and PC2 form a heteromeric complex primarily localized to the primary cilia, plasma membrane, and endoplasmic reticulum (ER) of renal epithelial cells (RECs), where they regulate calcium signaling, mechanosensation, and cellular homeostasis to prevent cystogenesis (2, 3, 4, 5). PC1, a large glycoprotein (>400 kDa), undergoes proteolytic cleavage at its N- and C-terminal domains, yielding fragments, including those from its ∼200-amino-acid C-terminal tail (CTT). The PC1-CTT is a focal point in ADPKD research due to the pathological high abundance of its cleavage products, such as the ∼30 kDa (PC1-p30) and ∼15 kDa (PC1-p15) fragments, in ADPKD kidneys and nonorthologous mouse models of polycystic kidney disease (PKD) (6, 7). Notably, controlled heterologous expression of a PC1-CTT fragment in a conditional *Pkd1* −/− mouse model can rescue cystic phenotypes, mirroring full-length PC1 and underscoring its functional significance (8). Soluble PC1-CTT fragments localize to diverse subcellular compartments, including mitochondria, nuclei, and lysosomes, influencing signaling and metabolic pathways (6). The PC1-CTT also harbors a coiled-coil domain (CCD) that mediates interaction with PC2’s C-terminal CCD, alongside localization signals for cilia, nuclei, and mitochondria (9). However, the roles of the intact, membrane-tethered PC1-CTT and its interplay with PC2 across cellular contexts remain poorly defined (10).

ADPKD is considered a ciliopathy, with PC1, PC2, and other cyst-associated proteins in the primary cilium playing central roles in cyst formation (1). Beyond cilia, PC1 is found in the plasma membrane, ER, and tight junctions, suggesting broader functions, while PC2 predominantly resides in the ER as a calcium-release channel but also localizes to cilia and the plasma membrane (11, 12). The phenotypic similarity of *PKD1* and *PKD2* mutations underscores the interdependence of PC1 and PC2 in ADPKD pathogenesis. Recent evidence highlights aberrant metabolic regulation, particularly lysosomal signaling and nutrient-sensing pathways, as key drivers of cystogenesis, yet the protein networks mediating these effects are incompletely characterized (13). Identifying PC1 and PC2 binding partners and their connections to dysregulated pathways, such as mammalian target of rapamycin (mTOR) and lysosomal positioning, remains a critical challenge. To address this, we employed BioID (proximity-dependent biotin identification) (14) to map the proximity interactomes of membrane-tethered PC1-CTT and PC2 under cycling and ciliated conditions. This study elucidates novel interactions, including lysosomal and nutrient-sensing complexes, providing a comprehensive resource to uncover mechanisms of ADPKD pathogenesis and identifing therapeutic targets.

## Experimental procedures

### Cell Lines and Culture Conditions

All reagents were from Sigma-Aldrich unless otherwise stated. The Flp-In human embryonic kidney (HEK) 293T-REx cell line (15) (Invitrogen) was maintained in Dulbecco's modified Eagle medium: high glucose (DMEM-high glucose; Sigma-Aldrich); supplemented with 10% fetal bovine serum (FBS), 2 mM L-glutamine (Froggabio), 100 U/ml penicillin, and l00 μg/ml streptomycin (Corning) antibiotic solution. The cells were maintained in a humidified incubator at 37 °C containing 95% air and 5% CO2 and grown as a monolayer in plastic tissue culture plates. The medium was refreshed every third day. Where required, ciliogenesis was induced by serum-depriving cells for 48 h in the described medium but with 0.5% FBS (16).

Human retinal pigment epithelium (RPE) hTERT *p53*^−/−^ Cas9 cells (kindly provided by Prof. Dan Durocher), murine inner medullary collecting duct (mIMCD-3) cells (Baltimore PKD center) were all grown in DMEM-F12 medium with 10% FBS. Normal human proximal tubule kidney (NHPTK) and ADPKD (Q4004X) telomerase-immortalized RECs (kindly provided by Prof. Robert Bacallao) were derived from normal human male kidney, and age and sex matched ADPKD kidneys respectively (17). NHPTK and ADPKD Q4004X RECs were grown in a DMEM-F-12 media containing growth hormones (epinephrine, hydrocortisone hemisuccinate, transferrin, Rh insulin, epidermal growth factor, triiodothyronine, and alanyl-L-glutamine), 10% FBS, and Amphotericin B.

BioID stable cell line generation was carried out as described previously (15, 16). Briefly, Flp-In HEK293 T-REx cells were plated in a six-well cell culture plate 18 to 24 h before transfection to achieve monolayer cells with 70∼80% confluency at the time of transfection. Constructs were co-transfected with Flp recombinase expressed from a pOG44 vector in 1:2 proportion using PolyJet (SignaGen Laboratories) transfection reagent. After 24 h, the medium was changed. On day two, the transfected cells were passaged to a 10 cm cell culture plate and selected with hygromycin B (200 μg/ml; BioShop) after 24 h. The selection media was changed every 3 to 4 days until clear visible colonies were present. The colonies were pulled, scaled up, and grown to 70% confluence before validation.

### Immunofluorescence and Western Blot

SDS-PAGE and western blotting was carried out as described previously (15, 18). Briefly, cell lysates were prepared in a lysis buffer containing 0.5 M Tris–HCl pH 6.8, 2.5 ml Glycerol, 2.0 ml 10% SDS, 0.2 ml 0.5% bromophenol Blue and protease inhibitor cocktail (EDTA-free protease inhibitor (Bioshop). Supernatant obtained by centrifugation at 5000 rpm for 5 min was resuspended in 4X Laemmli buffer and β-mercaptoethanol. Samples were separated with SDS-PAGE and transferred to nitrocellulose membrane (Bio-Rad). Immunoblot analysis was performed with the indicated antibodies and visualized with Clarity western ECL substrate (Bio-Rad). The signal intensities were analyzed using an imaging analyzer (Bio-Rad Chemidoc). Contrast and brightness adjustment was applied to the whole images and later quantified using Image J (NIH, USA, Version 1.54i).

The following primary antibodies were used for immunofluorescence (IF) experiments: Monoclonal antibodies raised against mouse anti-FLAGm2 (Sigma-Aldrich; 1:500) and PC1 7E12 (Santa Cruz; 1:500). Polyclonal antibodies used here were raised against BLOC1S1 (Proteintech; 1:500), BLOC1S2 (Novus; 1:100), DTNBP1 (Cusabio; 1:100), SNAPIN (Proteintech; 1:100), BORCS7 (Cusabio; 1:200), ARL13B (Proteintech; 1:500) and TCTN3 (Cusabio; 1:200). Streptavidin-488 (Abcam; 1:500) was used to detect biotinylated proteins in IF experiments and Streptavidin-HRP (Bio-Rad; 1:5000) was used to detect biotinylated protein in immunoblotting experiments; both without a secondary antibody. Secondary antibodies used for IF were pre-absorbed donkey anti-mouse or donkey anti-rabbit versions coupled to Alexa 488, 594, or 647 fluorophores (Invitrogen; 1:1000).

### Cyst and Tubulo-morphogenesis Assays

mIMCD-3 cells undergo cystogenesis when cultured in Matrigel (19). mIMCD-3 cells with mCherry-shRNA knockdown genes or scrambled control sequences were grown in Engelbreth-Holm-Swarm mouse sarcoma Matrigel (Corning). Cells were seeded at 500 to 1000 cells per 16 mm glass coverslip in a complete media. Media were changed every 2 days, and cyst growth was monitored till day 6. Cyst numbers were counted from five fields of view taken at 4x (0.13NA) on an EVOS FL imager (Thermo Fisher Scientific). Cyst size was measured using OrganoSeg (20). Images were resized and segmented on OrganoSeg with out of focus correction threshold set at 100 and intensity threshold set at 1. The cross-sectional area of each cyst was then masked to measure cyst area in pixels (1 pixel = 2.1 μm). For size quantifications, at least 15 cysts were captured per condition. All cyst experiments were repeated at least three times. Where indicated for use in cyst assays, mIMCD-3 cells harboring shRNAs were stably transfected with the CD16.7-PKD1 construct (PC1-CTT) and selected with G418 (250 μg/ml).

**Fig. 1:**
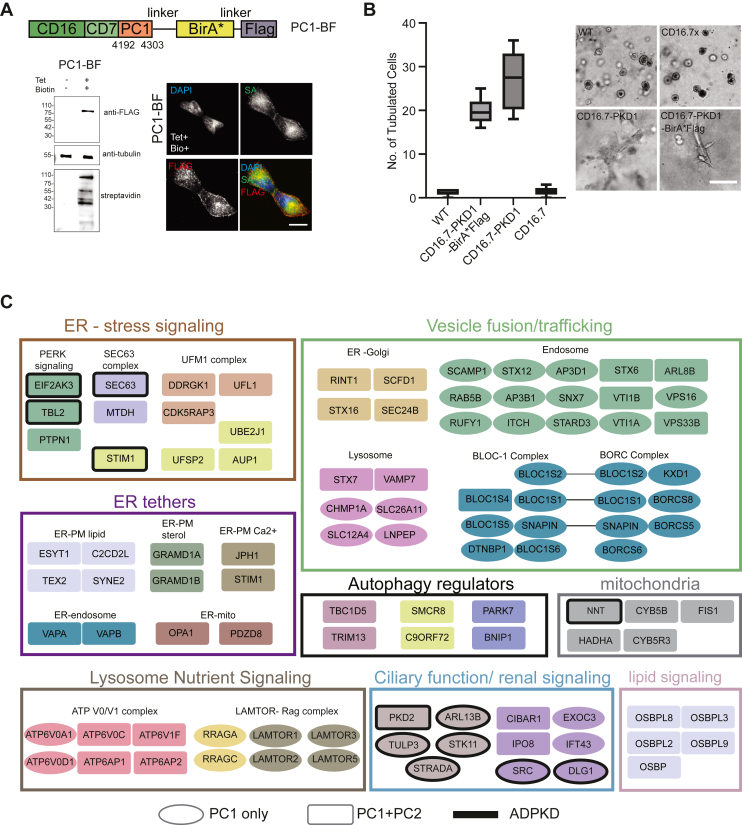
**PC1-BF interactome***.A*, *Top*: schematic representation of the CD16.7-PKD1-BirA∗-FLAG (PC1-BF) construct depicting the location of BirA∗ ligase and FLAG epitope with respect to the coding region of PC1. Residue numbers of the C terminal 112 amino acids of PC1 are denoted. *Bottom left*: western blot showing expression of PC1-BF (*left panel*) in HEK293 cells as detected by the anti-FLAG epitope (top blots), in the presence (+) or absence (−) of the inducer tetracycline (Tet). Antitubulin (*middle* blots) was used as a loading control, while the biotinylation activity of BirA∗ was inferred from probing the blots with streptavidin-HRP (bottom blots) in the presence of exogenous biotin. Reference molecular weight markers are indicated on the *left* of each blot, and the bait protein migrated at the expected molecular mass (PC1-BF ∼80 kDa). *Bottom right*: Representative *gray* scale micrographs of PC1-BF stable cell lines induced with tetracycline (Tet+) and treated with biotin (Bio+) for 24 h followed by fixation and immunofluorescence labeling. Panels depict nuclear (DAPI) and biotin labeling (fluorophore conjugated streptavidin, SA) and fusion protein (anti-FLAG) expression, with a pseudocolored merge image. The scale bar represents 20 μm. *B*, *Leftpanel*: Box-whisker plot of the extent of tubulation in collagen grown mIMCD-3 cells stably transfected with constructs CD16.7-PKD1-BirA∗-FLAG (encoding PC1-BF), CD16.7-PKD1 or CD16.7 alone. *Box* denotes 25 to 75 percentiles, and the *horizontal line* denotes the median of the data (pooled from six replicates, *n* = 200 cells total). *Right panel*: corresponding brightfield micrographs of fields of cells for each condition, as labeled. The scale bar represents 150 μm. *C*, Gene Ontology (GO) enrichment analysis was used to classify the cellular component terms of the PC1-BF high confidence interactors identified by BioID. Preys corresponding to genes known to associate with ADPKD are outlined in *bold black*, while preys present in both PC1-BF and PC2 datasets are represented as rectangles (see legend). ADPKD, autosomal dominant polycystic kidney disease; DAPI, 4′,6-diamidino-2-phenylindole; HRP, horseradish peroxidase; mIMCD, mouse inner medullary collecting duct; PKD, polycystic kidney disease.

**Fig. 2:**
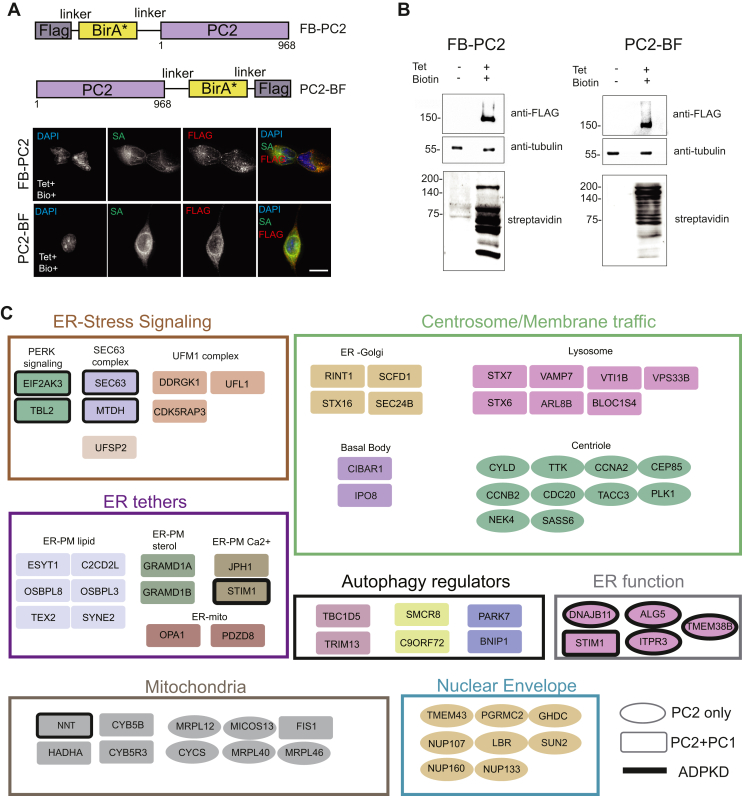
**PC2 interactome.***A*, *Top:* schematic representation of the PC2-BF and FB-PC2 constructs depicting the location of BirA∗ ligase and FLAG epitope with respect to the coding region of PC2. *Bottom*: representative *gray* scale micrographs of PC2-BF/FB-PC2 stable cell lines induced with tetracycline (Tet+) and zbiotin (Bio+) for 24 h followed by fixation and immunofluorescence labeling. Panels depict nuclear staining (DAPI), biotin labeling (fluorophore conjugated streptavidin, SA) and fusion protein (anti-FLAG) expression, with a pseudocolored merge image. The scale bar represents 20 μm. *B*, western blot showing expression of FB-PC2 (*left*) and PC2-BF (*right*) in HEK293 cells as detected by the anti-FLAG epitope (*top blots*), in the presence (+) or absence (−) of the inducer tetracycline (Tet). Antitubulin (*middle blots*) was used as a loading control, while the biotinylation activity of BirA∗ was inferred by probing with streptavidin-HRP (*bottom* blots) in the presence of exogenous biotin. Reference molecular weight markers are indicated on the *left* of each blot, and all baits migrated at the expected molecular mass (PC2-BF/FB-PC2 ∼145 kDa). *C*, Gene Ontology (GO) enrichment analysis was used to classify the cellular component terms of the PC2-BF/FB-PC2 high confidence interactors identified by BioID. For this analysis, the PC2-BF/FB-PC2 datasets were merged. Preys corresponding to genes known to associate with ADPKD are outlined in *bold black*, while preys present in both PC1-BF and PC2 datasets are represented as *rectangles* (see legend). ADPKD, autosomal dominant polycystic kidney disease; DAPI, 4′,6-diamidino-2-phenylindole; HRP, horseradish peroxidase.

**Fig. 3:**
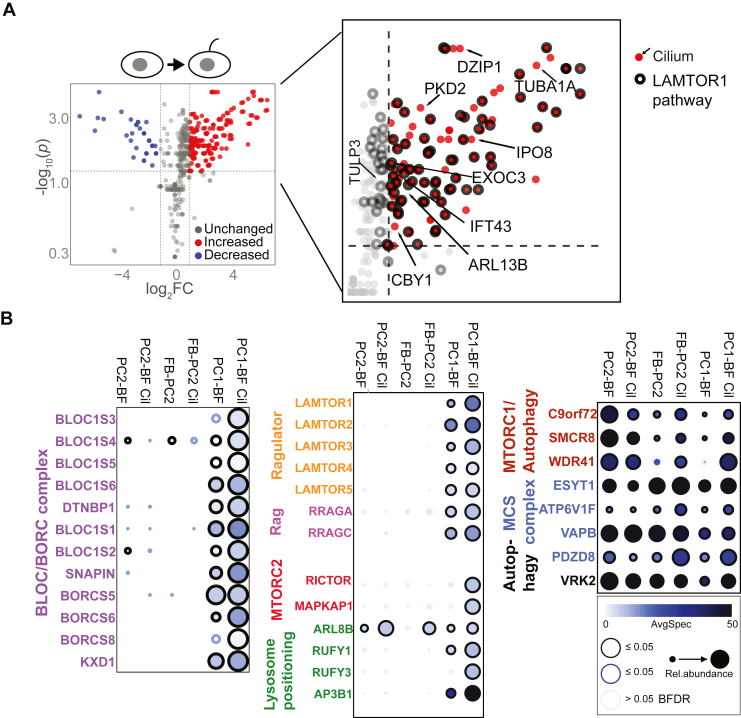
**Interactome changes during ciliation.***A*, volcano plot of significance *(y*-axis) *versus* fold change (*x*-axis), comparing biotinylated proteins identified in PC1-BF nonciliated *versus* ciliated BioID analyses. Fold change (FC) was calculated by comparing the average spectral count of each prey in the ciliated and nonciliated data sets. High confidence interactors with FC ≥ 2 (*vertical dotted lines*) spectral counts are indicated (*red*, increased; *blue*, decreased); significance threshold (*p*) < 0.05 (*horizontal dotted line*). Preys falling below thresholds are marked in *gray* (unchanged). Zoomed inset of the *top right* quadrant of the volcano plot highlights several enriched preys (in *red*) at or above threshold, with those known to be associated with primary cilia marked with *arrows*. A second enriched group of known LAMTOR1 network proteins is highlighted with outlined *circles*. *B*, dot plot of prey spectral counts of several ciliation-enriched preys from (*A*), and their abundance profiles across different baits: PC1-BF/PC2-BF/FB-PC2, and their ciliated states (Cil). Dot color scales according to average spectral count, and dot size scales according to its relative abundance with different baits. The border outline color scales according to its Bayesian false discovery rate (BFDR) probability (see legend and Experimental procedures).

**Fig. 4:**
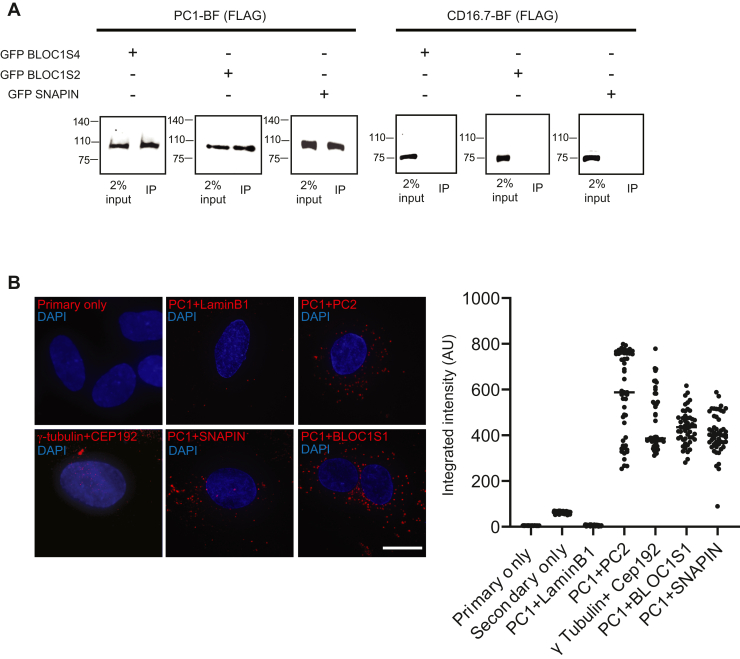
**Validation of PC1-BLOC-1/BORC interaction.***A*, coimmunoprecipitation of transiently transfected GFP-tagged BLOC-1 components (BLOC1S4, BLOC1S2, and SNAPIN) in cell lines stably expressing PC1-BF (*leftpanel*) or negative control CD16.7-BF (*right panel*). Cell lysates were incubated with anti-GFP beads and processed for western blotting with anti-FLAG antibodies. The FLAG epitope is present on both PC1-BF (∼90 kDa) and CD16.7-BF (∼75 kDa) constructs (see schematic in Fig. 1*A*). *B*, left panel shows representative pseudocolor merge micrographs of NHPTK RECs fixed and processed for a proximity ligation assay (PLA), depicting amplified fluorescence in the presence of interacting partners (red channel) and nuclei (DAPI). PLA was performed with background controls (primary only and secondary only), a negative control (PC1 + LaminB1), and a positive control (ɣ-tubulin + CEP192) and used to detect PC1+BLOC1S1 and PC1+SNAPIN association using endogenous antibodies. *Rightpanel* plot shows a representative quantification of the per-cell amplified PLA signal (*solid black dots*; *n* = 50 cells) under each condition, with the mean of the population denoted by a horizontal line. ∗∗ denotes *p* < 0.01 by Student’s *t* test between the specified population and the PC1 + LaminB1 negative control. The experiment was repeated three times with the same trends. The scale bar represents 20 μm. DAPI, 4′,6-diamidino-2-phenylindole.

**Fig. 5:**
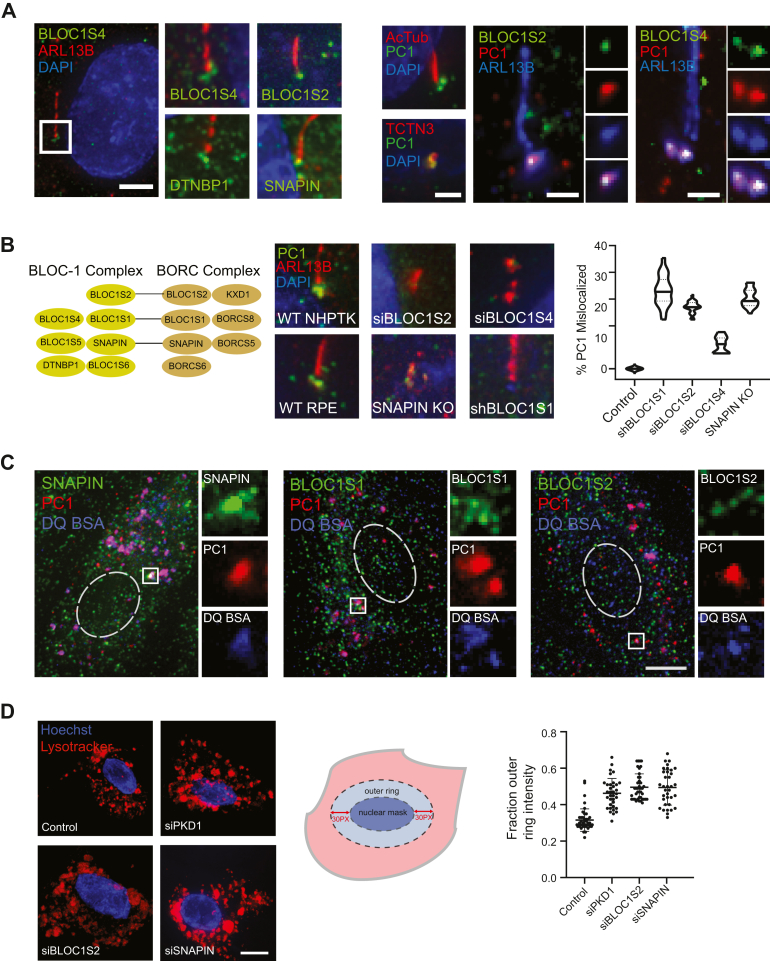
**Functional significance of BLOC-1/BORC.** (*A*, *left series*) Representative pseudocolored merge micrographs of immunofluorescence labeling in ciliated NHPTK RECs showing nuclear staining (DAPI), a cilia marker (ARL13B) and GFP-BLOC-1 protein fusions (BLOC1S4, BLOC1S2, DTNBP1, and SNAPIN). Cilia marked by ARL13B were magnified (*white box*) and shown in zoomed panels. The scale bar represents 10 μm, zoomed panels are 2.5x. (*A*, *right series*) Pseudocolored merge micrographs of ciliated NHPTK RECs immunolabeled for ciliary markers (Acetylated tubulin, AcTub; TCTN3, a transition zone marker; ARL13B) in parallel with anti-PC1 (*left panel*), or GFP-BLOC1S2 and PC1 (*middlepanel*), or GFP-BLOC1S4/PC1 (*rightpanel*). The cropped insets in the middle and *right panels* highlight the signal per channel in the basal body region of the cilium. The scale bar represents 10 μm. *B*, main panel shows representative micrographs of anti-PC1 and anti-ARL13B labeling upon depletion of BLOC-1 components in NHPTK RECs (siBLOC1S2/4 or shBLOC1S1) or knockout in RPE cells (SNAPIN KO), as compared to their respective wild-type (WT) controls. Scale is the same as in (*A*). Corresponding violin plot of the percentage of ciliated cells where PC1 was no longer present at the base of the cilium marked by ARL13B in each condition (*n* = 100 cells pooled from three experiments). Values are normalized to the WT control and are statistically different from control (*p* < 0.05 by Student’s *t* test). *Solid horizontal lines* in each condition denote the median, while dotted lines denote quartiles. *C*, representative pseudocolor merge micrographs of NHPTK cells processed for endogenous antibody immunolabeling of PC1, lysosomes (as marked by DQ-BSA), together with endogenous anti-SNAPIN (*left* panel), anti-BLOC1S1 (middle panel), and anti-BLOC1S2 (*right* panel). The *dashed white* ellipse overlay represents the approximate location of the nucleus in each cell. The *boxed white region* is zoomed 4x in the insets and individual fluorescence channels are shown to highlight the overlap. The scale bar represents 15 μm. *D*, *left panel* shows representative psuedocolor merge micrographs of live-imaged NHPTK RECs loaded with nuclear (Hoechst) and lysosomal (LysoTracker) marker probes after transfection with siRNAs against BLOC1S2, SNAPIN, or PKD1, as compared to mock transfected control (as labeled). The scale bar represents 20 μm. *Rightpanel* shows a representative quantification scatter plot of the fraction integrated intensity (of total per cell) corresponding to a 30-pixel wide ring outside of the nuclear mask (*dark blue* area) as defined in the cartoon (*light blue* area), that was measured (*n* > 35 cells) in each condition. *Horizontal solidlines* denote mean ± SD, and all siRNA treated conditions were significantly different from the mock transfected control (*p* < 0.01 by Student’s *t* test). The experiment was repeated thrice with similar trends. DAPI, 4′,6-diamidino-2-phenylindole; DQ-BSA, dye-quenched bovine serum albumin; PC1, polycystin 1 NHPTK, normal human proximal tubule kidney;; PKD, polycystic kidney disease; REC, renal epithelial cell; RPE, retinal pigment epithelium.

**Fig. 6:**
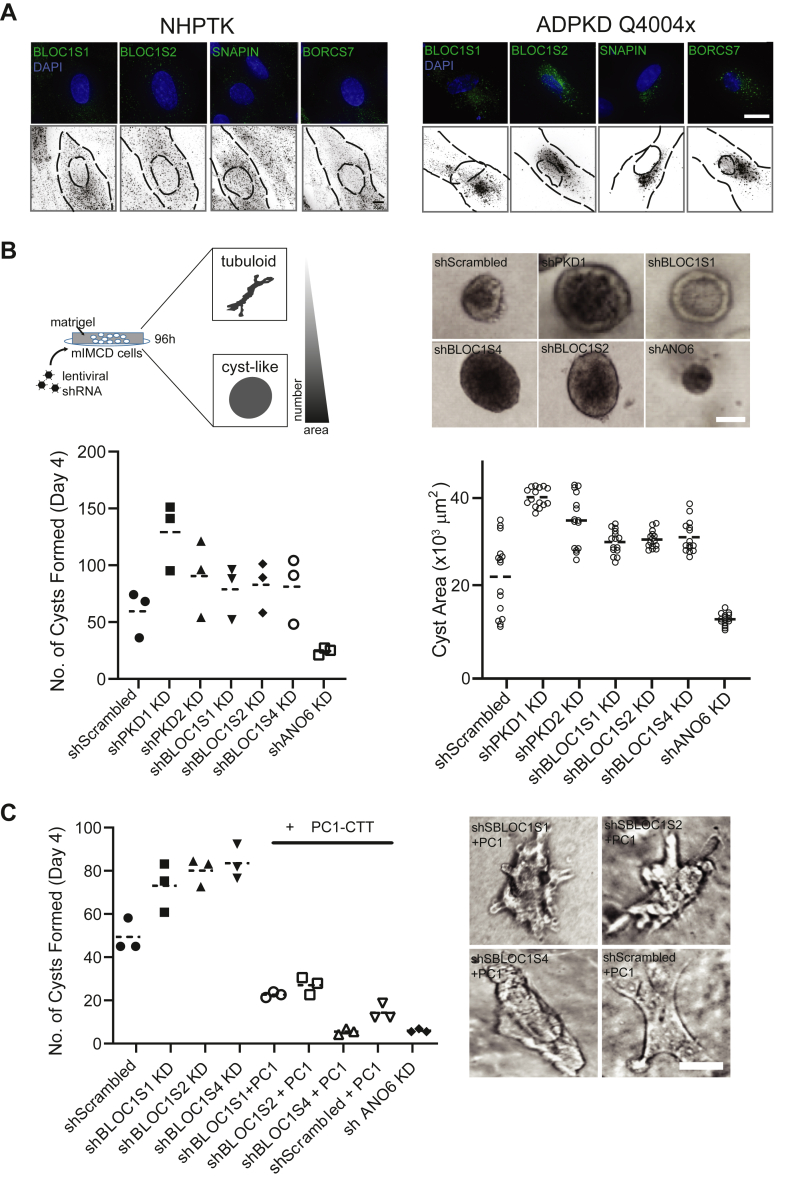
**Role of BLOC-1/BORC in cellular disease models of ADPKD.***A*, representative psuedocolor merge micrographs of immunofluorescence labeling in NHPTK (*leftpanels*) and ADPKD Q4004X (*rightpanels*) RECs showing nuclear (DAPI), and anti-BLOC1S1, -BLOC1S2, -SNAPIN and -BORCS7 labeling. An inverted *gray scale* image of the antibody channel is shown alongside for easier visualization of the BLOC-1/BORC subunit distribution. The cell and nuclear outlines for each cell are depicted with *black dashed lines*. The scale bar represents 15 μm. *B*, *Top left*: cartoon flowchart of the 3D cystogenic assay in mIMCD-3 cells. *Top right*: representative micrographs of phase contrast brightfield images of mIMCD-3 control (shScrambled KD) and shRNA knockdown cells seeded in Matrigel, which exhibited cyst growth over a 6-day period. The scale bar represents 150 μm. The number of cysts formed at day 4 (*leftlower panel*), and cyst areas (*right lower panel*) were quantified for each knockdown condition. Cyst numbers depicted as symbols, were counted for each condition (mean is shown as *dashed horizontal line* for each condition), and three replicate experiments are plotted. All mean values were significantly different from the control (*p* < 0.01 by Student’s *t* test). For cyst area, 15 cysts were randomly sampled from the pooled experiments and measured (see Experimental procedures). *C*, the cyst assay in (*B*) was repeated with control (shScrambled KD) or BLOC-1/BORC shRNA knockdown cells, additionally with or without stably transfected PC1-CTT (see Experimental procedures). Cells were then plated as in (*B*) and the number of cysts quantified (*leftpanel*) and representative micrographs were taken (*rightpanel*). The scale bar represents 45 μm. ADPKD, autosomal dominant polycystic kidney disease; BORC, BLOC-one-related-complex; CTT, C-terminal tail; DAPI, 4′,6-diamidino-2-phenylindole; mIMCD, mouse inner medullary collecting duct; NHPTK, normal human proximal tubule kidney; PC1, polycystin 1; REC, renal epithelial cell.

Tubulogenesis in mIMCD-3 cells was carried out with 3D Collagen Type I (2 mg/ml; Corning) as described previously (21), but without EGF and forskolin supplementation. Collagen was supplemented with Glutamax (24 mM), NaHCO_3_ (2.35 mg/ml), and Hepes (20 mM). mIMCD-3 cells stably expressing PC1-BF were selected using hygromycin (4 μg/ml) and CD16.7-PKD1, CD16.7 alone constructs were selected with G418 (250 μg/ml). Stable cell lines were seeded at 500 cells per 16 mm coverslip for tubulogenesis. Complete media without growth factors were changed every 3 days and tubulogenesis was monitored up to day 15.

### Lentiviral Transduction and Generation of Stable Cell Lines

To generate viral particles, 2 × 10^6^ HEK293-T (ATCC: CRL-3216) cells were plated in T75 flasks the day before transfection. Retroviral transfer plasmid (8 μg), VSVG (8 μg), and Pax5 (4 μg) were transfected into the cells with PolyJet (SignaGen Laboratories), according to the manufacturer’s instructions. Media supernatant containing viral particles were collected at 48 h and transduced into cells plated in 15 cm plates at 60% confluency. Multiplicity of infection was determined by plating cells to 40% confluency in 6-well plates and subjecting them to varying concentrations of viral particles (22). Polybrene sensitivity was determined by plating cells at 30% confluency with varying concentration of polybrene from 1-8 μg/ml for 1 week. Polybrene (6 μg/ml) was added to viral particles as per the calculated multiplicity of infection, prior to transduction. Polybrene-containing media were replaced with complete media the next day. For stable lines, mIMCD-3 cells that were transduced with shRNAs and hTERT RPE *p53*^−/−^ cells that were infected with SNAPIN-LentiCRISPR or PKD1-LentiCRISPR KO virus, selection was carried out with 20 μg/ml puromycin 48 h post transduction. mIMCD-3 knockdown cells were used for cyst assays and hTERT RPE *p53* −/− Cas9 CRISPR KO cells were screened for single clones and following validation experiments were used for the localization assay.

### DNA Cloning and Constructs

The CD16.7-PKD1-CTT-BirA∗-FLAG fusion construct, termed PC1-BF, and the CD16.7-BirA∗-FLAG control vector (CD16.7-BF) were generated by subcloning CD16.7-PKD1 and CD16.7 alone (23, 24, 25) into a C-terminal BirA∗ Flp-In vector (15, 16), respectively. CD16.7-PKD1 is a chimeric fusion construct which encodes the extracellular CD16 domain, the transmembrane CD7 domain, and the last C terminal and cytoplasmic 112 aa of PKD1 (23, 24, 25). The full-length 200 aa PC1-CTT has multiple organelle targeting sequences, including two ciliary targeting sequences (CTS (26, 27)), a nuclear targeting seqeunce (28), and two mitochondrial targeting sequences (MTS (6, 8, 29)). The 112 aa PC1-CTT sequence utilized for the BioID here lacks the putative NLS (LRRLRLWMGLSKVKEFRHKVR) and CTS (RHKVRFEG) motifs and retains one of the putative MTS (GLSVSLGRLGTRCEPEPSRLQAVFEA) and CTS (VHP) motifs as well as the CCD (AVFEALLTQFDRLNQQATEDVYQLEQQL) motif important for its retained interaction with PC2. Full length human PKD2 was fused C or N terminal into BirA∗-Flag expression vectors to generate PC2-BF and FB-PC2, by seamless cloning using the NEB HiFiBuilder kit (New England Biolabs). GFP-BLOC1S2, GFP-BLOC1S4, and GFP-SNAPIN constructs were ordered as complementary DNA clones from GenScript. shRNAs designed against PKD1, PKD2, BLOC1S1, BLOC1S2, BLOC1S4, ANO6, and guide RNAs against SNAPIN and PKD1/2 were procured as lentiviral cassettes from VectorBuilder.

### Gene Knockout (KO) and Knockdowns (KD)

To generate CRISPR KOs of SNAPIN, BLOC1S2, and DTNBP1, we utilized hTERT RPE *p53* −/− Cas9 cells (22). Cells were treated with 20 μg/ml puromycin for 48 h and cell viability counting was performed using a hemocytometer (22). Around 500 cells were seeded onto a 15 cm plate in a dropwise manner and left for a week to allow for single-cell colony formation. On day 4, well isolated colonies were picked using 4.8 mm disposable sterile cloning discs (Sigma-Aldrich). Subsequently, 20 to 25 clones were picked, and the discs were transferred to 24-well plates with complete media containing and then scaled up to 6-well plates over 2 weeks, following which they were harvested for western blot analysis for screening of positive clones. SiRNAs against BLOC1S2 and BLOC1S4 (Silencer select, Invitrogen) were transfected at 20 nM using RNAiMAX (Invitrogen) as per the manufacturer’s instructions. For siRNA controls, we used mock transfected RNAiMAX alone. Where ciliation was required, serum levels were changed to 0.5% FBS after 48 h of transfection to induce serum deprivation. Cells were harvested for functional assays after an additional 48 h (16). Mouse shRNAs corresponding to mCherry-Scrambled, mCherry-PKD1, mCherry-PKD2, mCherry-BLOC1S1, mCherry-BLOC1S2, mCherry-BLOC1S4, and mCherry-ANO6 for KD in mIMCD-3 cells and human BLOC1S1 shRNA for NHPTK RECs were procured from VectorBuilder, and were subjected to lentiviral transduction as described above. After 48 h of transduction, mIMCD-3 cells were selected using 6 μg/ml of puromycin and 20 μg/ml of puromycin for NHPTK RECs following three rounds of selection for 2 weeks. Validation of gene knockdown was performed using western blotting and IF.

### Imaging, Lysosomal and Proximity Ligation Assays

Imaging was carried out as described previously (15) with a widefield deconvolution fluorescence microscope (DeltaVision, GE Healthcare) using 60×oil immersion objective, NA 1.42. All exposure settings were identical within experiments. High resolution images were acquired as Z-stacks (0.2 μm × 50) and deconvolved, projected, and exported to 16 bit TIFFs into MATLAB or Image J with Fiji addon for further processing.

The lysosome probes LysoTracker Red (LTR) (1 μg/ml; Invitrogen) and the nuclear stain Hoechst 33342 (1 μg/ml) were used for live-cell imaging. Cells were plated in serum replete and serum starved conditions prior to 24 h in Lab-Tek 8-well chambers. Cells were treated with LTR for 30 min at 37 °C and washed again twice and imaged immediately. For dye-quenched bovine serum albumin (DQ-BSA) retention assays, DQ-BSA was used at 10 μg/ml (30), and cells were exposed for 16 h at 37 °C. Cells were then washed with PBS and fixed with 4% paraformaldehyde, mounted, and imaged as above. For quantification of lysosome integrated intensity in the outer band around the nucleus, we utilized the MATLAB image analysis tool kit (Mathworks Inc). We subtracted dark noise and background using demarcated regions in several images from each dataset. The background was calculated using the most populated pixel bin from histograms of these regions (for each channel) and subtracted from the corresponding channels. These were additionally processed with a Top-hat background correction transform to remove haze. Each image was cropped to generate single nuclei followed by adaptive thresholding to generate a nuclear mask. Each nuclear mask was dilated by 30 pixels and then the original mask was subtracted from it, resulting in a masked ring around the nucleus. The integrated intensity of all pixels within the ring for each cell was calculated and plotted.

Proximity ligation assay (PLA) (20) was performed using the Duolink Proximity Ligation Assay kit (Sigma-Aldrich) containing a plus and a minus probe, as per the manufacturer’s instructions. NHPTK RECs were plated on 16 mm coverslips under nonstarved conditions for 24 h and subsequently fixed with 4% paraformaldehyde. Cells were then subjected to 30 min of blocking solution followed by overnight incubation with primary antibodies (1 mg/ml) in antibody diluent solution. Plus and minus probes were also left overnight for conjugation in mouse and rabbit secondary antibodies (1 mg/ml), respectively. On day 2, the conjugated probe solution was applied to cells and later ligation and amplification steps were carried out, as per the manufacturer's protocol. Cells were then mounted and imaged as above. Image analysis (50 cells per condition) was carried out in Fiji/Image J as described (31), with thresholding and followed by calculation of integrated PLA signal per cell.

### Coimmunoprecipitation

HEK293 stably expressing PC1-BF and empty CD16.7-BF were transiently transfected with GFP-BLOC1S2, GFP-BLOC1S4. and GFP-SNAPIN using Lipofectamine 2000 (Invitrogen) and induced for 24 h with tetracycline (1 μg/ml) after transfection. Cells were then lysed in RIPA buffer (10 mM Tris/Cl pH 7.5, 150 mM NaCl, 0.5 mM EDTA, 0.1% SDS, 1% Triton X-100, and 1% deoxycholate) as per the manufacturer’s protocol (GFP Trap magnetic agarose, Proteintech). Lysates were clarified at 17,000 g at 4 °C for 10 min. Twenty-five microliters of bead slurry per experimental condition were equilibrated with dilution buffer (10 mM Tris/Cl pH 7.5, 150 mM NaCl, 0.5 mM EDTA) supplemented with protease inhibitor cocktail (Sigma-Aldrich) and washed twice in wash buffer (10 mM Tris/Cl pH 7.5, 150 mM NaCl, 0.05% Nonidet P40 Substitute, 0.5 mM EDTA) for 5 min at 4 °C and then equilibrated in lysis buffer for 5 min at 4 °C. Beads were incubated with clarified lysate for 1 h at 4 °C under rotating agitation. Following incubation, beads were washed three times for 10 min in wash buffer at 4 °C and then resuspended in 60 μl denaturing buffer. The beads were then boiled to release the bound proteins. Coimmunoprecipitation results were analyzed by western blotting using an anti-Flag antibody to detect PC1-BF.

### Proximity-Dependent Biotinylation

BioID and mass spectrometry (MS) were conducted as described previously (18). Cells were grown in five 15 cm cell culture dishes to 70% confluence, then incubated for 24 h in complete media supplemented with 1 μg/ml tetracycline (BioShop) and 50 μM biotin (BioShop). Cells were at 85 to 90% confluency prior to processing. Cells were lysed, sonicated twice for 10 s at 35% amplitude (Sonic Dismembrator 500; Thermo Fisher Scientific) and the resulting lysate centrifuged at 16,000 rpm (35,000*g*) for 30 min at 4 °C. Supernatants were passed through a Micro Bio-Spin Chromatography column (Bio-Rad 732–6204) and incubated with 30 μl of high-performance streptavidin-packed beads (GE Healthcare) for 3 h at 4 °C on an end-over-end rotator. Beads were collected (2000 rpm, 2 min) and washed six times with 50 mM ammonium bicarbonate (pH 8.3). Beads were then treated with L-1-Tosylamide-2-phenylethyl chloromethyl ketone (TPCK)-treated trypsin (Promega) for 16 h at 37 °C on an end-over-end rotator. Another 1 μl of TPCK-trypsin was added and incubated in a water bath at 37 °C for 2 h. Supernatants were lyophilized and stored at 4 °C for downstream MS analysis (16).

### MS and Data Analysis

Briefly, LC-MS/MS was conducted using a 120 min reversed-phase gradient (0–30% CH_3_CN in 0.1% HCOOH) running at 225 nl/min using a Easy nLC1200 pump in-line with a Q Exactive HF mass spectrometer (Thermo Fisher Scientific). A parent ion scan was performed using a resolving power of 60,000 fwhm. The 20 most intense MS1 peaks were selected for MS/MS (15,000 fwhm) and fragmented using higher energy collision-induced dissociation. For protein identification, .raw files were converted to the.mzXML format using Proteowizard (32), then searched using X!Tandem (33) and Comet (2018.01 rev. 4) against Human RefSeq Version 45 (containing 36,113 entries). Search parameters specified a parent MS tolerance of 10 ppm and an MS/MS fragment ion tolerance of 0.4 Da, with up to two missed cleavages allowed for trypsin. Oxidation, deamidation, and acetylation were allowed as variable modifications. Data were analyzed using the trans-proteomic pipeline via the ProHits 7.0.0 software suite (32), which applies filtering at the peptide level (Peptide Prophet, 5% false discovery rate [FDR]). Trans-proteomic pipeline was also applied at the protein level with iProphet using a probability cutoff of 0.9. Only proteins to which two or more unique peptides were assigned were considered for identification. Raw MS data are available at MSV000097854.

### BioID Experimental Design and Statistical Rationale

SAINTexpress v. 3.6.1 (33, 34) was used to score the probability that identified proteins were enriched above background. SAINTexpress uses a model whereby each prey identified in a BioID experiment with a given bait is compared using spectral counts as a measure of abundance, against a set of negative controls (15, 16). A minimum of two biological replicates were used for all analyses (PC1-BF, CD16.7-B, PC2-BF, and BF-PC2, under ciliated/serum deprivation and nonciliated/serum replete conditions, respectively). BioID datasets were highly reproducible, all replicates for each condition were tested for correlation and ensured to have an average R^2^ value > 0.9 before proceeding with the analysis. For running SAINTexpress, nine negative controls (five FlagBirA∗-empty nonciliated and four FlagBirA∗-empty ciliated) were compressed to four virtual controls, meaning that the four highest spectral counts for each identified protein were used in the scoring to increase stringency. Scores were averaged across both biological replicates, and these averages were used to calculate a Bayesian false discovery rate (BFDR); preys detected with a BFDR of ≤1% were considered high-confidence. For the PC1 bait, in addition to the FlagBirA∗ control, we performed two manual filtering steps to select a high confidence dataset. First, any prey also present in the PC2 dataset was included; and second, any preys enriched 4x or more in the CD16.7B dataset were excluded (see Supplementary Tables S1 and S2 for raw and filtered datasets, respectively).

### Bioinformatics and Data Visualization

Gene Ontology enrichments were performed using ToppGene (35). The PKD dataset included all high-confidence preys defined in the SAINT output file (Supplementary Table S1). The networks were generated using Cytoscape 79 version 3.8.0 (36). Dot plots were generated using ProHits-viz78 (37). Quantitation is encoded using the color gradient representing control-subtracted spectral counts (capped at 50), with relative spectral counts across baits represented by node size. Border color is encoded by BFDR value (black ≤0.01; blue ≤0.05; light blue >0.05). Prey profiles were compared to a curated BioID dataset of 192 cellular markers (38) and the Jaccard distances between datasets were exported for analysis and visualization in Microsoft Excel. Volcano plots were generated using VolcaNoseR (39) using thresholds of *p* < 0.05 and fold change cutoff range of ±2 ((15, 16). For evaluating ciliary categorization of preys, we used SYSCILIA Gold Standard v2 (40).

## Results

### Generation and Validation of PC1-CTT, PC2, and Control Lines

Membrane-tethered PC1-CTT, when heterologously expressed from the well-described CD16.7-PKD1 construct, retains several functional characteristics of intact PC1 (23, 24, 26) and allows for interaction with PC2 (41, 42, 43). The CD16.7.PKD1 construct was fused with a FLAG-tagged R118G mutant of the *Escherichia coli* BirA biotin ligase (44) (BirA∗) (Fig. 1*A*), and the chimeric sequence was targeted to the flippase recognition target locus in engineered Flp-In HEK293 T-REx cells to generate a BioID line (15). Stable lines were verified to express CD16.7.PKD1-BirA∗-FLAG (hereby referred to as PC1-BF) at the predicted size (Fig. 1*A*). In cycling cells, PC1-BF localized to the plasma membrane, and to numerous intracellular punctate structures (Fig. 1*A*). Biotinylation of the fusion protein was confirmed by IF and western blotting with streptavidin probes (Fig. 1*A*). Similar to untagged CD16.7-PC1, the PC1-BF construct was capable of inducing tubulation in a 3D murine *in vitro* model of tubulo-morphogenesis (19) (Fig. 1*B*). As controls, we utilized stable lines expressing BirA∗ alone (15) or empty CD16.7-BirA∗-FLAG (CD16.7-BF; Supplementary Fig. S1, *A* and *B*). Next, we generated stable cell lines expressing full-length PC2, fused either N or C terminally with FLAG-BirA∗ (FB-PC2 and PC2-BF, respectively). Both expression and biotinylation of the fusion proteins were confirmed (Fig. 2, *A* and *B*). IF labeling indicated that FB-PC2 and PC2-BF proteins localized mainly to the ER (Fig. 2*A*), which is the predominant location for native PC2 (5, 45). Cycling cells expressing PC1-BF, FB-PC2, PC2-BF, or control protein “baits”, were induced and supplemented with biotin for 24 h before processing for BioID (15). Identified interactions with prey proteins were analyzed for significance using SAINTexpress (34), with high-confidence interactors (“preys”) defined as those with a FDR of ≤1% (0.01).

### Global Overview of PC1-B and PC2 Interactomes

The PC1-BF interactome comprised 507 interactors (Supplementary Table S2). FB-PC2 yielded 379 interactions, 257 (∼70%) of which were also present in PC2-BF (Supplementary Fig. S1*C*; Supplementary Table S2). In addition, 266 of the 507 PC1-BF preys (∼50%) were also present in the PC2-BF or FB-PC2 datasets. The predictive power of our approach was supported by the identification of 18 interactors previously associated with PC1/2 or renal cystic disease phenotypes (documented in Supplementary Table S3). Six of these proteins were previously reported PC1 interactors (PKD2 (46), NNT (8, 47), SEC63 (48), STK11 (LKB1), STRADA (49), and SRC (50); Fig. 1*C*), and five are previously reported PC2 interactors (TBL2 and EIF2KA3 (51, 52), STIM1, ITPR3 (53) and TMEM38B/TRIC-B (54); Fig. 2*C*). Deletion/mutation of two additional PC1-BF interactors (TULP3 and ARL13B) results in the development of PKD in preclinical models (55, 56, 57), and two additional PC1-BF-specific interactors (DLG1 and CASK), have been linked to renal cystic development (58, 59). Another two PC2 preys identified here (DNAJB11 and ALG5) were recently classified as atypical ADPKD genes linked to processing and trafficking defects (60). A PC1-BF-specific interaction with GPCR5A, which was recently demonstrated to be a marker of renal cystic epithelial cells (61, 62), was identified in this analysis. Finally, an additional overlap of 10 interacting preys was noted when compared to a proteomic study of PC1 interacting proteins published previously (47).

Gene Ontology enrichment and Cell Map (38) analysis revealed that PC1-BF interactors are localized to the ER, lysosomal, mitochondrial, and vesicle membranes (Fig. 1*C*; Supplementary Fig. S1*D*). Enriched functional categories include autophagy regulators, lipid signaling, ER stress signaling, ER-tethering, and vesicle fusion and trafficking (Supplementary Fig. S1*D*). PC2 interactors were also enriched for ER and mitochondrial-associated functions, as well as lysosomal and autophagy regulators (Figs. 1*C* and 2*C*). Categories unique to PC2 included mitochondrial and nuclear envelope membrane proteins (primarily identified by FB-PC2), and centrosome/mitotic proteins (primarily identified by PC2-BF) (Fig. 2*C*). When compared with other recently published proximity interactomes (38), preys from PC2-BF and FB-PC2 overlapped substantially with those from ER resident, multipass bait proteins like DERL-1 and SEC61 (tagged at their cytosol facing termini; Supplementary Fig. S1*D*). It is likely that the PC2-BF and FB-PC2 interactomes represent cytosolic facing associations, as we note significant overlap with N-terminal BirA∗ tagged ER protein LRRC59 (which is cytosolic) but not its luminal counterpart (C-terminal BirA∗-tagged LRRC59; Supplementary Fig. S1*D*). By contrast, the membrane tethered PC1-BF interactome overlaps largely with those of membrane trafficking proteins (Supplementary Fig. S1*D*).

Consistent with earlier reports demonstrating the preferential mitochondrial targeting of PC1-p15 via a MTS (6), and the interaction of PC1-p30 with the mitochondrial protein, NNT (8), it was anticipated that PC1 baits might identify mitochondrial preys. Indeed, NNT was identified in both PC1 and PC2 interactomes. Comparison of the BioID dataset against human MitoCarta 3.0 (63) (Supplementary Table S7) revealed that ∼10% (116/1136) of annotated mitochondrial proteins were represented in the entire dataset.

### Enriched PC1-BF Interactome Under Ciliation-Induced/Serum Deprivation Conditions

BioID was also conducted in the same cell lines maintained for 48 h in reduced serum (0.5%) to induce ciliation (16) (Fig. 3*A*; Supplementary Fig. S2*A*). A number of additional high confidence PC1-BF interactors were identified under these conditions, including the known cilia components/regulators (40) ARL13B, TULP3, DLG1, DZIP1, IPO8, IFT43, TUBA1A, EXOC3, and PKD2. Three additional ciliary components (LRRC49, CIBAR1 (FAM92A), and CBY1) that form a complex at the transition zone at the base of the cilium (64), were identified in the PC2 interactome (Supplementary Fig. S2*B*). IPO8 is a member of a subclass of nuclear import adapter proteins (importins) and may mediate ciliary cargo trafficking through the transition zone (65). We also observed that several PC1-BF preys enriched in the ciliation-induced/serum deprivation state were associated with the LAMTOR1 network (39) (Fig. 3*A*; Supplementary Tables S5 and S6). This included all components of the BLOC-1 complex, which is found on tubular endosomes and plays an important role in the biogenesis of lysosome-related organelles by facilitating cargo sorting (40). We also identified seven of the eight subunits of the BORC complex (Figs. 1*C*, 3*B*; Supplementary Table S2). BLOC-1 and BORC share three components (BLOC1S1/BLOS1/BORCS1, BLOC1S2/BLOS2/BORCS2, and SNAPIN/BLOC1S7/BORCS3) and BORC regulates the positioning of lysosomes at the cell periphery (39). In this context, PC1-BF also associated with components of the Ragulator-Rag complex (LAMTOR1-5, RRAGA, and RRAGC) and an additional five enriched preys linked to lysosomal positioning (ARL8B, DENND6A, RUFY1, RUFY3, and AP3B1 (66)) (Fig. 3*B*). Finally, we detected several members (VPS16, VPS33A/B, STX17, VPS18, VPS8, and VIPAS39) of the homotypic fusion and vacuole protein sorting (HOPS) and class C core endosomal vacuole tethering (CORVET) complexes (67). The HOPS/CORVET complexes interact with Rab-GTPases and SNARE proteins to regulate vesicle transport, fusion, and maturation in autophagy and endocytosis pathways (67, 68, 69).

A global overview of the preys common to PC1-BF, FB-PC2, and PC2-BF under ciliated/nonciliated conditions, revealed that the majority were associated with ER, endosomal, lysosomal, and Golgi membranes (Supplementary Fig. S3*A*), and functional categories included autophagy regulators, lipid and sterol binding, ER stress signaling, ER-tethering, and vesicle fusion and trafficking. The top 15 cilia-enriched preys for FB-PC2/PC2-BF datasets respectively, are tabulated here (Supplementary Fig. S3*B*).

Given the identification of several lysosomal-associated preys in the BioID dataset, we performed an extensive prey comparison against several large lysosomal datasets (70, 71, 72) to determine the relative prevalence of lysosomal proteins, which we have detailed here (Supplementary Tables S4–S6). This analysis revealed a significant enrichment of lysosomal preys in the PC1-BF *versus* PC2 datasets across all the databases examined (PD 2.3, 17% *versus* 5%, Supplementary Table S4*b*; hLGDB, 11% *versus* 5%, Supplementary Table S5*b*; Akter et al., 2023, 17% *versus* 8%, Supplementary Table S6*b*, respectively). Across the dataset comparisons, 73 preys were identified in at least two lysosomal databases with 28 (33%) present in both PC1-BF and PC2, 37 (51%) in PC1-BF only, and 8 (11%) in PC2 only datasets. Of the 35 lysosomal preys identified as being lysosomal in all three databases, 14 (40%) were present in both PC1-BF and PC2 datasets, 18 (56%) were PC1-BF-specific, and three (9%) were PC2-specific.

In regard to the BLOC-1/BORC complex, only BORC, and not BLOC-1, components were identified as being lysosomal in at least two databases with all preys identified being PC1-BF-specific (Supplementary Table S4*a*). Finally, this analysis revealed the interaction of PC1-BF/PC2 with six subunits of the lysosomal V-ATPase, which is known to associate with the Ragulator-Rag and BORC complexes (73).

### BLOC-1/BORC Interaction With PC1

We next focused on the significance of the novel PC1 interaction with the BLOC-1/BORC complex, and its relevance to PKD biology. The association of BLOC-1/BORC components with PC1 was validated using two orthogonal methods. First, three heterologously expressed BLOC-1/BORC components (GFP-BLOC1S4/-BLOC1S2/-SNAPIN) could coimmunoprecipitate PC1-BF, but not CD16.7-BF (Fig. 4*A*). To rule out the possibility of overexpression artefacts from heterologously expressing BLOC-1/BORC subunits, we validated antibodies to BLOC1S1/2 and SNAPIN using CRISPR-mediated knockout or shRNA based knockdown cell lines of the respective subunits (Supplementary Fig. S4). These validated antibodies were then used in a PLA with PC1 and BLOC1S1/SNAPIN in NHPTK RECs (Fig. 4*B*). A positive PLA reaction was observed with PC1 when paired with BLOC1S1 or SNAPIN, but not when paired with a negative control (LAMINB1), or conducted with no primary antibody (Fig. 4*B*). As positive controls for PLA, we utilized endogenous antibodies directed against two established markers that associate at the centriole (CEP192 and TUBG1). Using PLA, we additionally verified the control PC1-PC2 and PC1-ARL13B interactions (Fig. 4*B* and Supplementary Fig. S2*C*, respectively), which were also detected by BioID (Fig. 1*C*).

### Functional Significance, and Colocalization of BLOC-1/BORC Components With PC1 at Cilia and Lysosomes

We next examined the localization and functional significance of the BLOC-1/BORC complex in NHPTK RECs, as well as in a ciliated cell model (RPE) line. GFP-tagged BLOC1S2/S4, DTNBP1/BLOC1S8/dysbindin and SNAPIN were expressed in ciliated NHPTK RECs and colabeled with ciliary markers (antibodies to ARL13B or acetylated-tubulin). In addition to punctate structures throughout the cell, all four BLOC-1 components localized to the base of the cilium (Fig. 5*A*). In these cells, endogenous PC1 was also detected at the ciliary base using a validated antibody (Supplementary Fig. S4; Fig. 5*A*), and colocalized with the transition zone marker TCTN3 (Fig. 5*A*) and GFP-BLOC1S2/S4 (Fig. 5*A*).

In NHPTK RECs depleted of BLOC1S1/2/4 with siRNA, or in a SNAPIN KO line (Fig. 5*B*), we noted lower levels of ciliation (data not shown). The majority of cilia (as marked by ARL13B), were significantly shorter than controls and/or displayed abnormal morphology (90% siBLOC1S1, 95% SNAPIN KO, and 75% siBLOC1S2, n > 100 cilia counted; Fig. 5*B*). Where intact cilia could be detected, endogenous PC1 localization to the ciliary base was also significantly reduced compared to control (Fig. 5*B*). Here, depending on the BLOC-1 component depleted, PC1 was either absent (BLOC1S1/S2/S4) from the base of the cilium, or displaced from the base to the ciliary axoneme (SNAPIN; Fig. 5*B*).

Endogenous labeling of PC1 in NHPTK RECs revealed the presence of numerous punctate structures in the cytoplasm. When endogenous PC1 was colabeled in combination with BLOC1S1/2 and SNAPIN (using antibodies as validated above), respectively, we also detected several punctate sites of colocalization in lysosomal compartments, as marked by dequenched BSA (DQ-BSA; see Experimental procedures) (Fig. 5*C*). Therefore, a fraction of PC1 is also present with BLOC-1/BORC components at lysosomes.

We next examined the significance of PC1 in regulating lysosomal morphology by using high-resolution live imaging of LTR-loaded NHPTK RECs that were depleted of PC1 or the BLOC-1/BORC components BLOC1S2/SNAPIN. As expected, depletion of BLOC1S2/SNAPIN resulted in reduced lysosomal distribution to the cell periphery, and increased clustering and fusion events in the perinuclear area (74). We quantified this positioning defect by measuring the total intensity of LTR-positive structures in the perinuclear region, which was defined as a ring of constant width around the nucleus (Fig. 5*D*). Notably, similar to that observed with depletion of BLOC-1/BORC components, depletion of PC1 also resulted in a clustered lysosomal phenotype (Fig. 5*D*), consistent with the same phenotype we previously observed in *Pkd1* KO mouse embryonic fibroblasts (MEFs) (13).

### Functional Relevance of BLOC-1/BORC Components in ADPKD and 3D Mouse Cyst Models

To assess the functional relevance of BLOC-1/BORC to ADPKD, we first examined the endogenous distribution of these components in patient derived kidney cells. Immortalized RECs from an ADPKD patient harboring the truncation mutation Q4004X (which removes the entire PC1-CTT (17)) were fixed and labeled with antibodies to BLOC1S2/S4, SNAPIN, and BORCS7 and compared to NHPTK RECs as controls. All four proteins exhibited abnormal perinuclear clustering in ADPKD Q4004X RECs as compared to NHPTK RECs (Fig. 6*A*).

Next, we tested the ability of BLOC-1/BORC subunits to drive cystogenesis in a 3D cyst assay. mIMCD-3 cells favor the formation of 3D cysts instead of tubular morphologies when plated on Matrigel (75, 76), and are routinely used as cystic models for ADPKD (77). mIMCD-3 cells were transduced with lentiviral shRNA constructs targeting control (scrambled), PKD1, PKD2, and BLOC1S1/2/4 and plated on Matrigel over 6 days. ANO6 was found in our dataset, and is a known regulator of cyst lumen formation in ADPKD (78), and was thus utilized here as a control. Control cells exhibited some cystogenesis under these conditions (mean ˜ 53 cysts per 500 cells plated), and this was reduced significantly in cells depleted of ANO6 (mean ˜ 18 cysts; Fig. 6*B*). Cells depleted of PKD1/2 (mean ˜ 123; 84 respectively) and BLOC1S1/2/4 (mean ˜ 73;77;75 respectively) formed significantly more cysts compared to those depleted with scrambled shRNA control or ANO6 shRNA (Fig. 6*B*). Cross-sectional area analysis showed that cysts formed in control and ANO6 depleted cells were smaller (∼22,500 μm^2^ and ∼12,700 μm^2^, respectively) than those formed in cells expressing PKD1/2 shRNA (∼40,900 μm^2^; 35,500 μm^2^ respectively) or BLOC1S1/2/4 shRNA (∼30,500 μm^2^; 31,100 μm^2^; 31,700 μm^2^, respectively).

To test whether the cystogenic effect of the knockdown of BLOC-1/BORC components was dominant over PKD1, we generated double stable mIMCD-3 lines expressing both BLOC-1/BORC shRNAs and a membrane-tethered PC1-CTT construct. We found that the presence of heterologously expressed PC1-CTT drove cells towards tubular structures (Fig. 6*C*), even if they were depleted of BLOC-1/BORC subunits. Thus, the cystogenic effect of knockdown of BLOC-1/BORC components can be ameliorated by the PC1-CTT.

## Discussion

Our BioID results establish a comprehensive proximity interactome for membrane tethered PC1-CTT as well as for PC2 under cycling and ciliated cell conditions. The large number of preys obtained for PC bait proteins can be rationalized by the identification of stable as well as transient interactions as well as the integration of these interactions over an extended labeling time. The experimental strategy is supported by the identification of several known, as well as predicted (based on their involvement with ADPKD progression or cytogenesis), interactors of PC1 and PC2 that we have documented here. Our BioID interactome data identifies 143 proteins that are shared between PC1-CTT and PC2 baits under all conditions tested (Supplementary Table S4), defining a significant shared interactome. This shared interactome is anticipated given the documented interaction between PC1 and PC2 via their respective C-terminal cytoplasmic CCD. The latter represents over half the PC1-CTT proximity landscape, and constitutes primarily ER tethers and stress proteins, lysosomal and autophagy regulators, and to a lesser degree, vesicle trafficking, lipid signaling and mitochondrial proteins. We posit that these interactors will be impactful starting points for delineating the cellular functions of PC1 and PC2.

These data complement recent studies that employed other proteomic (47, 79) and transcriptomic (80, 81) methods to catalogue PC1 interactions, albeit with different cellular contexts, bait design and target isolation strategies. The soluble PC1-p15 form of cleaved PC1-CTT (which closely resembles the PC1-BF tail portion in this study), has been reported to be overexpressed in ADPKD cells and tissues (6). It will be therefore important to determine the relative proportions and functions of uncleaved (membrane-tethered) and cleaved PC1 forms in different cell types, and under different cellular states. We conclude that the PC1-BF interactome reported here will be a valuable benchmark for assessing differences between datasets generated with full-length (or other portions of) PC1 protein (82). That our PC1-BF dataset contains numerous instances of already validated PC1 interactions supports the utility and biological relevance of the PC1-CTT.

The PC2 data presented here, generated by fusion with the BirA∗ moiety at either cytosolic end, represents the first global view of the interaction landscape of this protein. As expected, a significant fraction of its proximal interactions constitutes structural ER membrane components. The ER is a pervasive, continuous tubular membrane network, which through its rapid peripheral dynamics can sample an entire cell volume within minutes (83). This pattern of mobility also constitutes frequent interorganelle tethering with mitochondria, lysosomes, lipid bodies and so on, which often signify distinct ER subdomains both functionally and structurally (83). PC2 has been reported to act as an ER stress sensor protein (5), to affect ER structure and ER-mitochondria tethering (84) and regulate potassium and calcium ion homeostasis (54). Our BioID data are consistent with these proposed roles and provides a starting point for molecular studies to investigate these mechanisms further. Notably, conformation specific BioID of PC2 identified several cell cycle progression and centrosome/basal body interactors (with the C-terminal BirA∗), while the N-terminal BioID was enriched with nuclear envelope and mitochondrial partners. In renal cells, the polycystin proteins localize on the centrosome and are important to maintain centrosome integrity (85), and centrosomal defects are associated with cystogenesis (86), although the mechanisms are unclear. Since PC1 and PC2 associate at their C termini via their respective coiled-coil domains (46), and the HEK293 cells in our study have low levels of endogenous PC1 (87), the availability of C-terminal PC2 interactors may be biased toward those that are normally shielded by the presence of PC1. Hence it will be very worthwhile to examine and compare these novel candidate interactions in renal cell and cystic models.

Recent findings have demonstrated a role of aberrant metabolic regulation as a driver for cystogenesis and cellular growth in ADPKD and other renal ciliopathies (88). Cyst-lining epithelia in several models of polycystic disease have been shown to have an activated lysosomal signaling pathway normally associated with nutrient deprivation and stress (13). The latter inputs trigger the activity of the mTOR sensor kinase and its target transcription factor EB (TFEB), and lead to lysosomal biogenesis, lysosome repositioning, and increased autophagic flux. Our PC1-BF data show striking enrichment of LAMTOR pathway components, particularly the lysosomal positioning BLOC-1/BORC complexes, in ciliation-induced/serum deprived cells. This enrichment appears to be specific to the PC1-BF bait *versus* the PC2 baits and was not observed with over 60 BioID experiments performed in ciliation-induced/serum deprived cells with baits at the centrosome/basal body or cilia (15, 16). In addition, BLOC-1/BORC preys were rarely observed in a large 192 bait pan-organellar BioID study (38).

Our validation and functional experiments suggest that there is a close association between PC1-CTT and the BLOC-1/BORC complex, although the precise nature of this association remains unclear. This is not without precedent, as the knockdown of mouse BLOC-1/BORC complex components pallidin (BLOC1S6) and dysbindin (BLOC1S8) in mouse cells has been shown to affect ciliogenesis and trafficking of specific membrane proteins, including PC2 (89). Our results showing colocalization of PC1 with BLOC-1/BORC components at the ciliary base (a site traversed by recycling endosomes (90)), and the observation that knockouts or knockdowns of BLOC1S2/S4/SNAPIN lead to delocalization of PC1 is consistent with a trafficking role for this complex. Interestingly, loss of function pallidin (BLOC1S6) and dysbindin (BLOC1S8) in *Dtnbp1*^*sdy/sdy*^ and *Pldn*^*pa/pa*^ mice, respectively, gave rise to significant dilations in collecting duct and proximal tubules of the kidney (89), which is consistent with our finding of increased cystogenesis in an *in vitro* cyst model. A second parallel consideration is a lysosomal role for PC1 through its BLOC-1/BORC complex association. This is supported by our findings that (1) a subpopulation of PC1 colocalized with BLOC-1/BORC components at lysosomal sites; (2) BioID with PC1-CTT identified 65 proximity associations with proteins identified as lysosomal (as compared to (70)), including the BLOC1/BORC complex, most of which were further enhanced in the ciliation-induced/serum deprivation state; (3) PC1 knockdown in proximal renal tubule cells resulted in abnormal lysosomal juxtanuclear clustering/morphology, which was also seen with BLOC-1/BORC depletion, and supports our recent data demonstrating a juxtanuclear clustering of the lysosomal compartment in *Pkd1*^−/−^ *versus* wild-type MEFs (13); 4) our observation that the Q4004X (PC1-CTT deleted) ADPKD cell line exhibits abnormal BLOC-1/BORC clustering.

In the context of the PC1-CTT interactome we describe, a plausible interpretation of our previous findings (13) documenting juxtanuclear lysosomal clustering in the absence of PC1 might be due to its interaction with the BLOC-1/BORC-ARL8B complex, which is important for lysosomal positioning. Therefore, it is important to note that while the PC1-CTT fragment (C terminal 112aa) utilized in this study lacks some known targeting sequences and regulatory elements, it retains a CCD that is crucial for its interaction with PC2 (6) and all eight BORC components contain CCDs, which are important for the complex to adopt a rod-like structure (91). These data present the possibility that PC1-CTT might interact with BORC via their mutual CCDs, which is currently under investigation.

A second, nonmutually exclusive possibility is that the phenotypes observed here are reminiscent of mutations or dysregulation of folliculin (FLCN), the primary protein involved in Birt-Hogg-Dubé (BHD) syndrome, which disrupts lysosomal positioning and mTORC1 signaling (92). Indeed kidney-specific deletion of either *Flcn* (93, 94) or both members of its associated complex, *Fnip1* and *Finp2* (95), results in renal cystic disease associated with constitutive Tfeb nuclear localization and alterations in mTORC activity, which in *Flcn* KO mice is completely rescued in the context of *Tfeb* KO (96). Therefore, the highly significant interaction of PC1-CTT and PC2 with the synonymous tripartite C9orf72:SMCR8:WDR41 complex (97) is an important observation given its structural parallel to the FLCN:FNIP1/2 complex (98). Thus, while no interactions with FLCN or FNIP1/2 were detected in our BioID experiments, it is plausible that the C9orf72:SMCR8:WDR41 complex performs an analogous yet distinct function via its interaction with polycystins. This hypothesis is particularly intriguing given that SMCR8, but not FLCN, contains an N-terminal CCD that interacts with other CCD proteins (99). It has further been shown that C9orf72 KO induces juxtanuclear clustering of lysosomes (100), supporting results reported herein and previously observed in *Pkd1* KO MEFs (13). It will therefore be very informative to further examine the interplay between the PC1-CTT and nutrient signaling pathways, especially with respect to the functioning of the BLOC-1/BORC complex.

In summary, the data presented here identifies a preferential interaction of PC1-CTT with a multimeric protein megacomplex known to be localized at the lysosome and involved in nutrient sensing and response. These data also provide the first PC1-CTT/PC2 BioID-based interactome that identifies a rich set of interacting proteins that can be further mined for potential biological significance and function and has the potential to uncover novel proteins involved in PKD pathogenesis.

## Data Availability

Raw MS data are deposited in the MassIVE repository (MSV000097854).

## Supplemental Data

This article contains supplemental data (70, 71, 72, 73).

## Conflict of Interest

The authors declare no competing interests.

## Supplementary data

Supplementary Figures Supplementary Figures 1. (*A*) Top: Schematic representation of the CD16.7-BirA∗-FLAG construct (CD16.7-BF) depicting the location of BirA∗ ligase and FLAG epitope with respect to the membrane tether CD16.7. *Bottom*: representative grayscale micrographs of CD16.7-BF cell lines induced with tetracycline (Tet+) and biotin (Bio+) for 24 hrs followed by fixation and immunofluorescence labeling. Panels depict nuclear (DAPI), biotin labeling (fluorophore conjugated streptavidin, SA) and fusion protein (anti-FLAG) expression, with a pseudo-colored merge image respectively. Bar = 20 μm. (*B*) Western blots showing expression of CD16.7-BF control, labeled as in (*A*). GAPDH was used as loading control. Western blot showing expression of CD16.7-BF in HEK293 cells as detected by the anti-FLAG epitope (top blot), in the presence (+) or absence (–) of the inducer tetracycline (Tet). Anti-GAPDH (middle blot) was used as a loading control, while the biotinylation activity of the cell lines was inferred from probing the blots with streptavidin-HRP (bottom blot) in the presence of exogenous biotin. Reference molecular weight markers are indicated on the left of each blot, and the bait protein was of the expected size (CD16.7-BF ∼70kDa). (*C*) Area proportional Venn diagram of the extent of overlap in preys identified in the PC1-BF, PC2-BF and FB-PC2 BioID interactomes in this study. (*D*) Comparison of PC1-BF (*top*), PC2-BF and FB-PC2 (*bottom*) interactomes with those from 192 BioID baits representing all major cellular compartments (38). Each table outlines the top 11 pairwise Jaccard Distance between the query bait and the comparison cellular marker bait profiles and is color-coded for similarity according to the descending scale on the left of each table. Lower similarity is associated with higher Jaccard distance between the bait pairs. For PC2 baits, an additional row representing a low similarity bait (LRRC59_Cterm) has been added for comparison. Supplementary Figure 2. (*A*) *Left panel* : representative pseudo-colored merge micrographs of immunofluorescence labeling in ciliated HEK stable lines used for BioID, showing nuclear labeling (DAPI), a cilia marker (ARL13B) and FLAG expression of the corresponding fusion construct (CD16.7-BF, PC1-BF, PC2-BF and BF-PC2) respectively. Scale bar = 5 μm. *Right panel*: histogram of ciliation frequency (mean± SD) in the respective BioID cell lines (n>500 cells counted in three independent experiments). (*B*) Dot plot of spectral counts of preys primarily associated with PC2-BF and FB-PC2 baits, grouped according to Gene Ontology (GO) cellular location or process. (*C*) Left panel shows representative pseudo-color merge micrographs of NHPTK RECs fixed and processed for a proximity ligation assay (PLA), depicting amplified fluorescence in the presence of interacting partners (*red channel*) and nuclei (DAPI). PLA was performed with background controls (primary only, see Fig. 4*B*) and used to assess enrichment of PC1+ARL13B association using endogenous antibodies in non-ciliated and ciliated (cil) cells. Right panel plot shows a representative quantification of the per-cell amplified PLA signal (solid black dots; *n* > 30 cells) under each condition, with the mean of the population denoted by a horizontal line. The ciliated PC1+ARL13B PLA signal was statistically significant with respect to the non-ciliated PC1+ARL13B and primary only control (Student’s t-test, *p*<0.01). Scale bar = 20 μm. Supplementary Figure 3. (*A*) Cytoscape software was used to generate a network representing high confidence preys (Bayesian false discovery rate <=0.01) that were shared between the PC1-BF, and one or both of PC2-BF/FB-PC2 baits (shown as solid black circles) in non-ciliated/ciliated cells. Each node represents a selected prey, organized by known localization and function through Gene Ontology enrichment analysis (see Materials and Methods) focused on statistically enriched cellular components and biological processes. Shared nodes shown with black outlines were also present in the non-ciliated state, while those without black outlines were ciliation-enriched. Two groups of nodes within the endoplasmic reticulum (ER) are colored and grouped according to their specific sub-compartments/processes (nuclear envelope, or ER contact site proteins). (*B*) Volcano plot of significance (y-axis) *versus* fold change (x-axis), comparing biotinylated proteins identified in FB-PC2 (l*eft panels*) and PC2-BF (*right panels*) non-ciliated vs ciliated BioID analyses. Fold change (FC) was calculated by comparing the average spectral count of each prey in the ciliated and non-ciliated data sets. High confidence interactors with FC ≥ 2 (vertical dotted lines) spectral counts are indicated (*red*, increased; *blue*, decreased); significance threshold (*p*) <0.05 (horizontal dotted line). Preys falling below thresholds are marked in gray (unchanged). *Bottom* panels show dot plots of prey spectral counts of several ciliation-enriched preys from the respective volcano plots, and their abundance profiles across the PC2-BF/FB-PC2 baits, and their ciliated states (Cil). Dot color scales according to average spectral count, and dot size scales according to its relative abundance with different baits. The border outline color scales according to its Bayesian false discovery rate (BFDR) probability (see legend and methods). Supplementary Figure 4. (*A*-*D*) Validation of BLOC-1/BORC component antibodies using CRISPR knockout (BLOC1S2, SNAPIN, DTNBP1) and knockdown (BLOC1S1) cell lines generated for this study. Western blot (*left panels*) clone screening and corresponding immunofluorescence labeling (*right panels*) showing endogenous BLOC-1/BORC component expression in RPE (*A*, *C*, *D*) or NHPTK RECs (*B*). For RPE cells, the knockout CRISPR clone chosen is denoted by ‘∗’ and a red box outlines its corresponding Western blot lane, showing the absence of endogenous antibody labeling compared to the wild type control lane, or to the clones that were negative for knockout (‘-’). The expected size of BLOC-1/BORC component is labeled on the right of the blots. In the *right* panels, the endogenous antibodies were validated using the knockdown or knockout of each gene product followed by immunofluorescence detection, as compared to the wild-type control. Grayscale fluorescence micrographs show representative fields of control (*top panels*) and knockdown (shRNA)/knockout (CKO clone∗) cells labeled for nuclei (DAPI) and the target BLOC-1/BORC component respectively. Scale bar = 150 μm.
