# Supplementary material for: Interaction Proteomics of Polycystins 1 and 2 Reveal a Novel Role for the BLOC-1/BORC Lysosomal Positioning Complex
Source: Mol Cell Proteomics. 2025 Oct 12;24(11):101091. doi: 10.1016/j.mcpro.2025.101091 (PMC12663635; doi:10.1016/j.mcpro.2025.101091)

Supplementary Figure 1

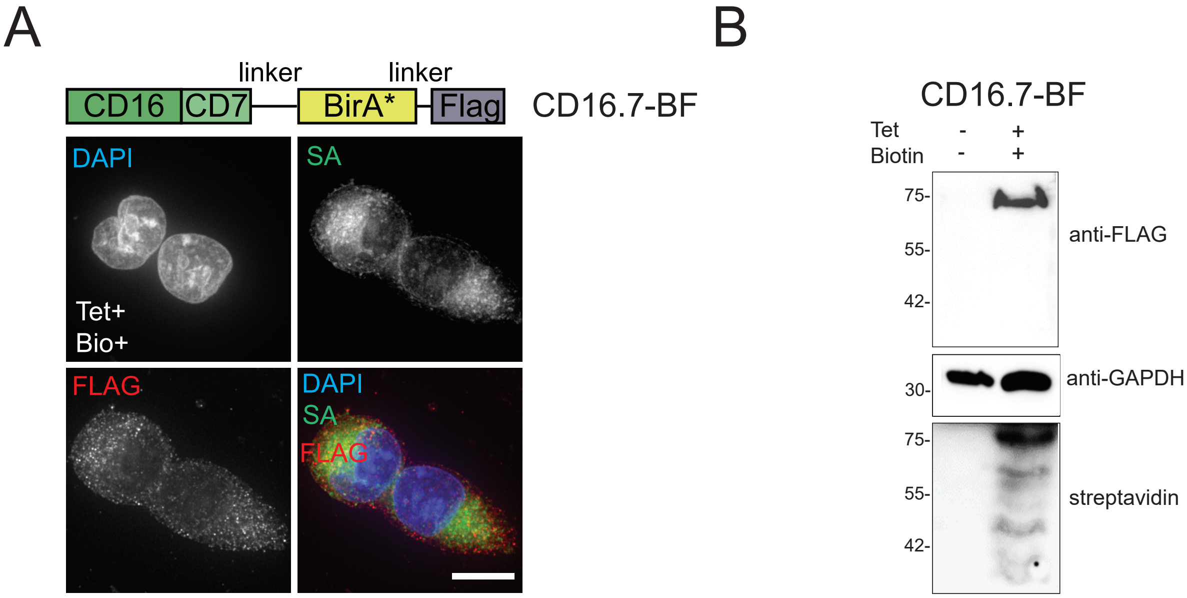

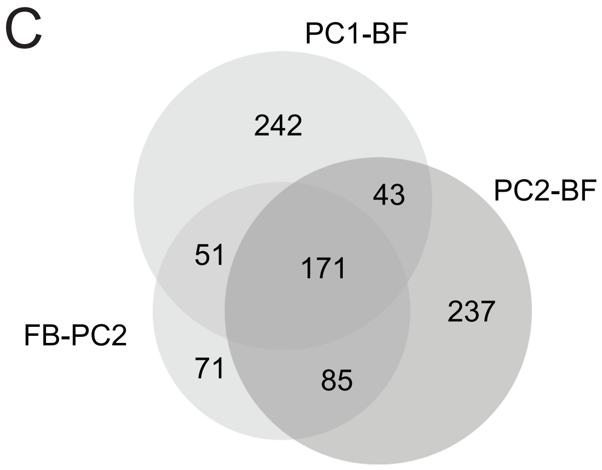

D

similarity

Bait vs PC1-BF	Distance	Intersection
RAB9A	0.739	171
RAB5A	0.748	147
STX7	0.756	164
METTL7A	0.763	137
RAB4A	0.781	128
STX4	0.784	124
RAB11A	0.786	146
STX6	0.789	120
GJD3	0.79	145
B3GAT1	0.794	127
GJA1	0.794	131

similarity

Bait vs PC2-BF	Distance	Intersection
SEC61B_Nterm	0.717	159
DERL1	0.736	140
EMD	0.736	141
ATP2A1	0.737	140
HSD17B11	0.738	141
CKAP4	0.743	120
SEC62	0.743	145
LRRC59_Nterm	0.744	126
METTL7A	0.747	145
RPN2	0.748	131
SSR1	0.751	128
LRRC59_Cterm	0.939	32

similarity

Bait vs FB-PC2	Distance	Intersection
SEC61B_Nterm	0.679	187
SEC62	0.702	174
DERL1	0.703	165
ATP2A1	0.706	164
LRRC59_Nterm	0.713	149
RPN2	0.713	156
HSD17B11	0.716	161
SSR1	0.72	151
RPN1	0.727	159
METTL7A	0.728	164
EMD	0.728	155
LRRC59_Cterm	0.914	48

Supplementary Figure 2

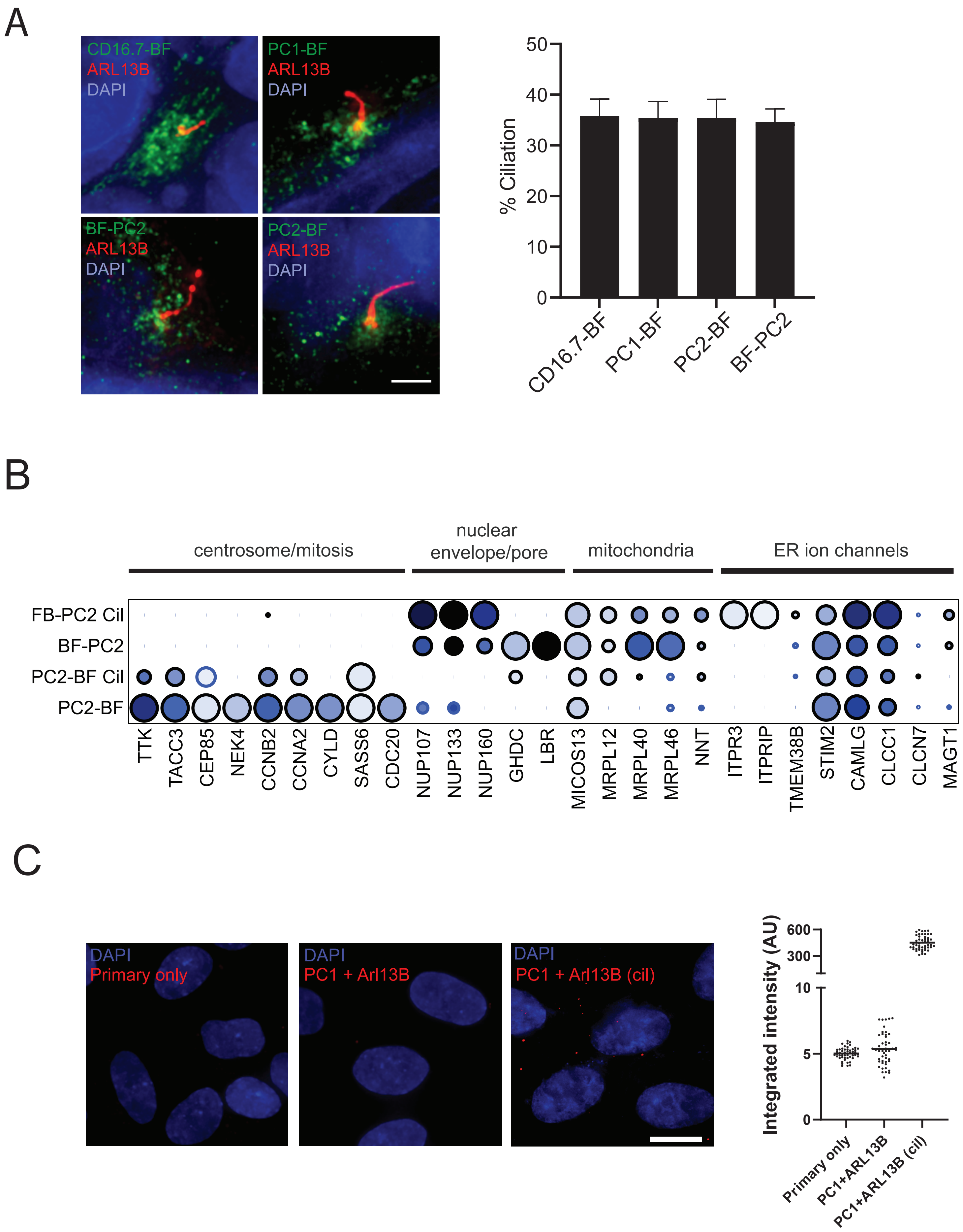

Supplementary Figure 3

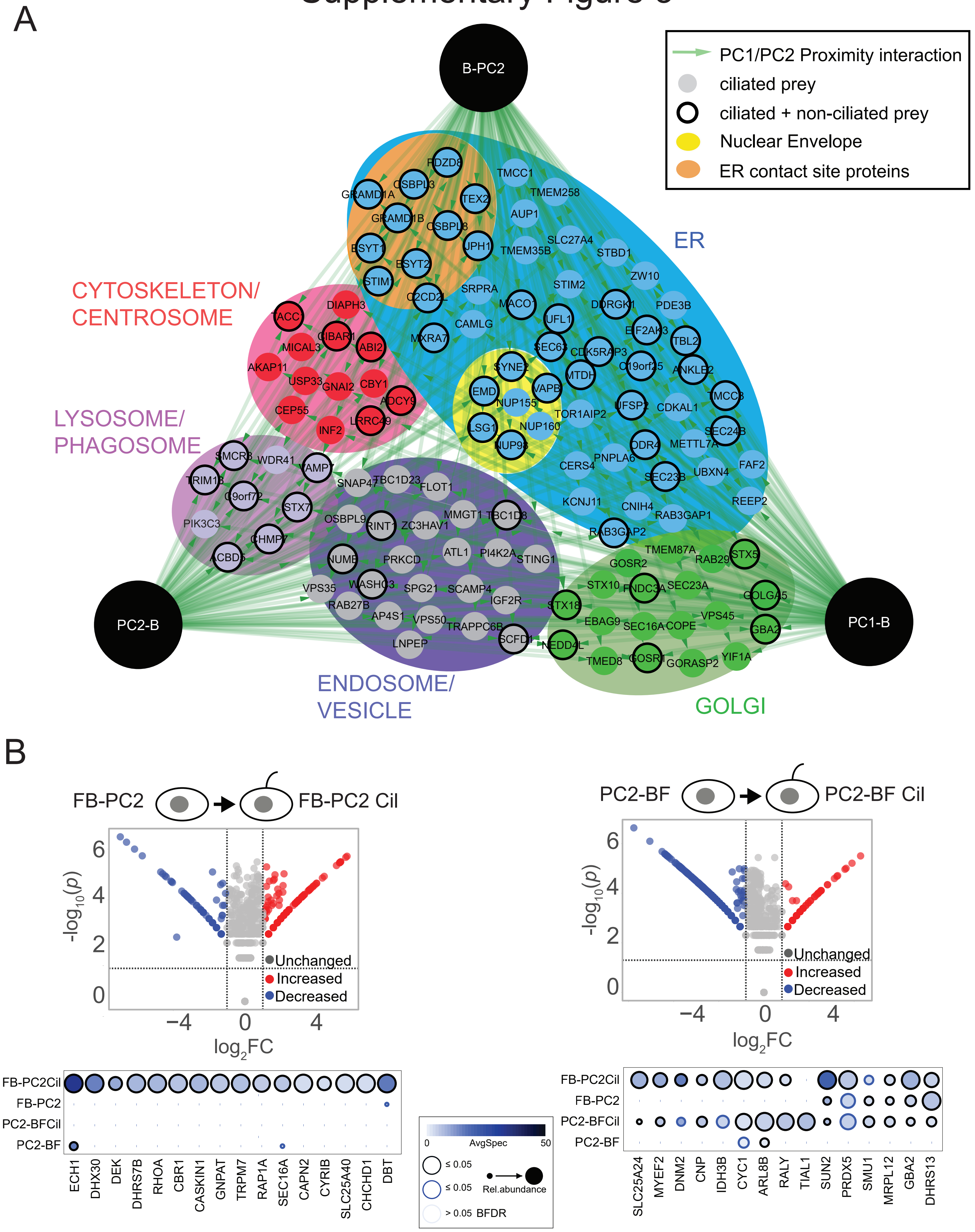

Supplementary Figure 4

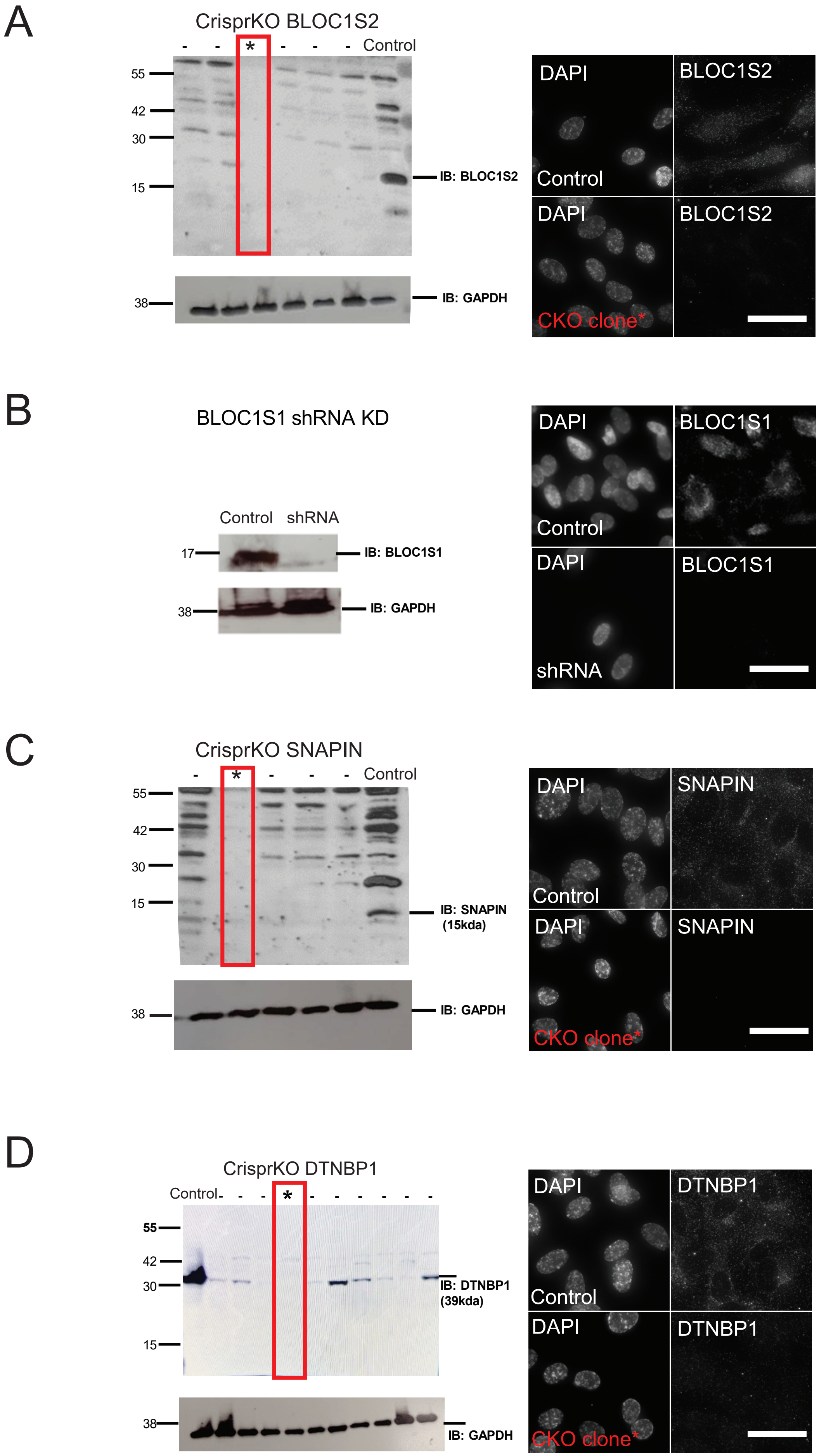

Supplement: Supplementary Figures — 1. (A) Top: Schematic representation of the CD16.7-BirA∗-FLAG construct (CD16.7-BF) depicting the location of BirA∗ ligase and FLAG epitope with respect to the membrane tether CD16.7. Bottom: representative grayscale micrographs of CD16.7-BF cell lines induced with tetracycline (Tet+) and biotin (Bio+) for 24 hrs followed by fixation and immunofluorescence labeling. Panels depict nuclear (DAPI), biotin labeling (fluorophore conjugated streptavidin, SA) and fusion protein (anti-FLAG) expression, with a pseudo-colored merge image respectively. Bar = 20 μm. (B) Western blots showing expression of CD16.7-BF control, labeled as in (A). GAPDH was used as loading control. Western blot showing expression of CD16.7-BF in HEK293 cells as detected by the anti-FLAG epitope (top blot), in the presence (+) or absence (–) of the inducer tetracycline (Tet). Anti-GAPDH (middle blot) was used as a loading control, while the biotinylation activity of the cell lines was inferred from probing the blots with streptavidin-HRP (bottom blot) in the presence of exogenous biotin. Reference molecular weight markers are indicated on the left of each blot, and the bait protein was of the expected size (CD16.7-BF ∼70kDa). (C) Area proportional Venn diagram of the extent of overlap in preys identified in the PC1-BF, PC2-BF and FB-PC2 BioID interactomes in this study. (D) Comparison of PC1-BF (top), PC2-BF and FB-PC2 (bottom) interactomes with those from 192 BioID baits representing all major cellular compartments (38). Each table outlines the top 11 pairwise Jaccard Distance between the query bait and the comparison cellular marker bait profiles and is color-coded for similarity according to the descending scale on the left of each table. Lower similarity is associated with higher Jaccard distance between the bait pairs. For PC2 baits, an additional row representing a low similarity bait (LRRC59_Cterm) has been added for comparison. Supplementary Figure 2. (A) Left panel : re [file mmc1.pdf]
